# Anti-Infective Bacteriophage Immobilized Nitric Oxide-Releasing Surface for Prevention of Thrombosis and Device-Associated Infections

**DOI:** 10.1021/acsabm.4c01638

**Published:** 2025-02-03

**Authors:** Vijay Singh Gondil, Morgan Ashcraft, Sama Ghalei, Anil Kumar, Sarah N. Wilson, Ryan Devine, Hitesh Handa, Elizabeth J. Brisbois

**Affiliations:** † School of Chemical, Materials and Biomedical Engineering, College of Engineering, University of Georgia, Athens, Georgia 30602, United States; ‡ Pharmaceutical and Biomedical Sciences Department, College of Pharmacy, University of Georgia, Athens, Georgia 30602, United States

**Keywords:** antibiotic resistance, nitric oxide, bacteriophages, thrombosis, bacterial infections, medical devices, and biomedical applications

## Abstract

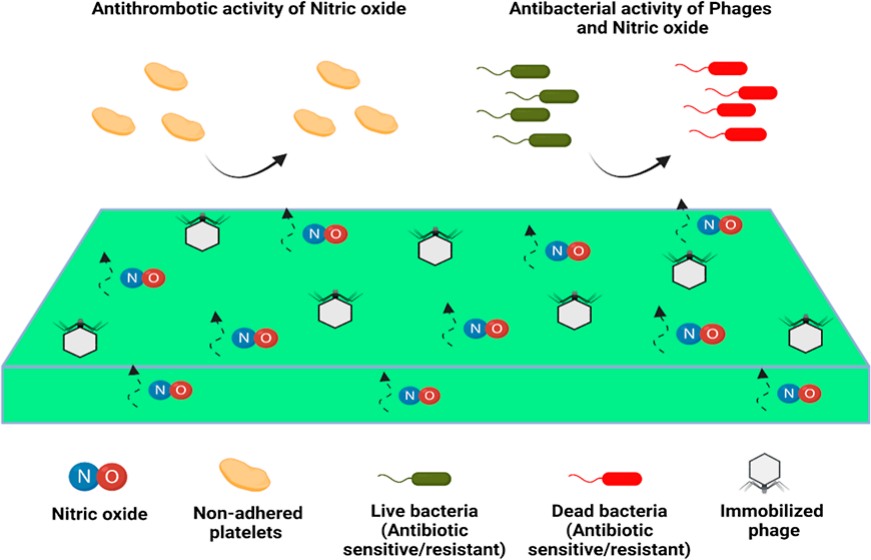

The treatment of critically ill patients has made great strides in the past few decades due to the rapid development of indwelling medical devices. Despite immense advancements in the design of these devices, indwelling medical device-associated infections and thrombosis are two major clinical problems that may lead to device failure and compromise clinical outcomes. Antibiotics are the current treatment choice for these infections; however, the global emergence of antibiotic-resistance and their biofilm formation abilities complicate the management of such infections. Moreover, systemic administration of anticoagulants has been used to counter medical device-induced thrombosis, but a range of serious adverse effects associated with all types of available anticoagulants entails exploring alternative options to counter device-associated thrombosis. In this study, bacteriophages (phages) were covalently immobilized on polydimethylsiloxane (PDMS) surface containing the nitric oxide (NO) donor *S*-nitroso-*N*-acetylpenicillamine (SNAP) via SNAP impregnation method. This dual strategy combines the targeted antibacterial activity of phages against bacterial pathogens with the antibacterial–antithrombotic activity of NO released from the polymeric surface. The PDMS, SNAP-PDMS, phage-immobilized PDMS (PDMS-Phage), and phage-immobilized SNAP-PDMS (SNAP-PDMS-Phage) surfaces were characterized for their surface topology, elemental composition, contact angle, SNAP loading, NO release and phage distribution. SNAP-PDMS and SNAP-PDMS-Phage surfaces showed similar and consistent NO release profiles over 24 h of incubation. Immobilization of whole phages on PDMS and SNAP-PDMS was achieved with densities of 2.4 ± 0.54 and 2.1 ± 0.33 phages μm^–2^, respectively. Immobilized phages were found to retain their activity, and SNAP-PDMS-Phage surfaces showed a significant reduction in planktonic (99.99 ± 0.08%) as well as adhered (99.80 ± 0.05%) *Escherichia coli* as compared to controls in log killing assays. The SNAP-PDMS-Phage surfaces also exhibited significantly reduced platelet adhesion by 64.65 ± 2.95% as compared to control PDMS surfaces. All fabricated surfaces were found to be nonhemolytic and do not exhibit any significant cytotoxic effects toward mammalian fibroblast cells. This study is the first of its kind to demonstrate the combinatorial pertinence of phages and NO to prevent antibiotic-resistant/sensitive bacterial infections and thrombosis associated with indwelling medical devices.

## 1 Introduction

Indwelling medical devices play a pivotal role in treating critically ill patients in hospital settings. Urinary and intravenous catheters (peripheral and central) are some of the most common types of indwelling devices and are frequently applied to most long-term hospitalized patients.^1^ The use of indwelling devices in medical care not only reduces morbidity and mortality but can improve the quality of life for many patients. However, the introduction of these medical devices into the body is also associated with a high risk of device-associated infections. Approximately 60–70% of nosocomial infections are related to indwelling medical devices or implants.^2^ Mortality from indwelling medical devices-associated infections is highly variable and device-dependent but ranges from less than 5% (such as Foley catheters) to more than 25% (such as mechanical heart valves).^3^ Medical device infections are usually initiated with the colonization of microorganisms followed by the formation of biofilm on the abiotic surfaces of implantable medical devices.^4^

Biofilms are 3-dimensional structures of microbial communities enclosed in a self-generated, protective extracellular matrix and attached to a solid substratum.^5^ Compared to planktonic cells, biofilms are 10–1000 times more resistant to conventional antibiotic treatments through multifactorial defense strategies such as poor penetration of antibiotics, formation of persister cells, slow growth, and nutrition depletion.^6^ According to the National Institute of Health (NIH), biofilms are responsible for approximately 80% of microbial infections and more than 60% of hospital-acquired infections, including device-associated infections.^7^ These infections significantly increase morbidity, mortality, length of hospital stay, and treatment costs. The cost of each device-associated infection may scale up to $30,000, particularly when mechanical ventilation is required along with admission to the intensive care unit and an extended hospitalization of 2–3 weeks.^8^ Dissemination of biofilms may also spread pathogens to the surrounding tissue or blood, establishing systemic bloodstream infections and resulting in worsened clinical outcomes.^9^ Antibiotic therapy is the treatment of choice to counter device-associated bacterial infections. However, the emergence of antibiotic-resistant bacterial pathogens and their biofilm-forming abilities complicated the management of device-associated infections. Antibiotic-resistant bacteria and biofilms are extremely recalcitrant to conventional antibiotic treatment and require high doses of antibiotics, which increases the rate of antibiotic resistance development and confers dose-associated toxicity to the host cells.

Along with bacterial colonization, medical implants also face challenges from the host’s system in terms of protein adsorption, complement cascade activation, and platelet aggregation, resulting in medical device-induced thrombosis.^10^ Thrombosis is one of the major complications in the use of blood-contacting medical implants, as it can lead to device failure through occlusion.^11^ To prevent medical device-induced thrombosis, anticoagulant, and/or antiplatelet therapies are the current clinical solutions, but these treatments also increase the risk of uncontrolled hemorrhaging and do not consistently prevent device-induced thrombosis.^12,13^ As such, there is an unmet need for novel biomaterials that prevent bacterial infections and exhibit antithrombotic activities in applications such as blood-contacting device materials. Antithrombotic material development has led to the introduction of nitric oxide (NO) donors in medical-grade polymers to address device-associated thrombosis.^14^ NO is a highly reactive, small, endogenous gas molecule involved in various biological functions, including vascular homeostasis, anti-inflammation, and neurotransmission.^15^*S*-nitroso-*N*-acetyl penicillamine (SNAP) is one of the most widely used NO donors because it provides physiological levels of NO release and its ability to integrate with medical-grade polymers via various methods.^16−18^ Nitric oxide donors (such as SNAP and *S*-nitrosoglutathione) incorporated within medical-grade polymers have demonstrated effectiveness in reducing bacterial viability as well as preventing both activation and adhesion of platelets.^19,20^ However, one concern that has been difficult to address is the balance between high levels of NO that lead to potent and efficient bacterial eradication, but may also exhibit cytotoxic effects on mammalian cells when NO levels are too high.^21^ Alternative antibacterial moieties can be combined with NO donors within or on the surface of medical device-grade polymers to maintain the balance between antibacterial activity, antithrombotic activity, and host biocompatibility. NO-releasing materials can be surface-modified as their surface properties remain unchanged even after the incorporation of NO donors into their polymer matrix.^22^ Therefore, the antibacterial activity of NO-releasing materials could be further increased by the surface immobilization of alternative antibacterial agents while retaining their antithrombotic effects.

Bacteriophages (phages) are considered potential alternatives to conventional antibiotics in treating bacterial infections.^23^ Phages are viral predators of bacteria, having a natural ability to recognize, infect, and lyse bacterial cells. The intrinsic antibacterial activity of phages against their host bacterial cells makes them fascinating candidates to counter antibiotic-resistant infections, which are refractory to conventional antibiotic treatment. Along with antibacterial activity, phages have other properties that make them more advantageous than commonly used antibiotics, including their high host specificity, self-amplification ability, no collateral damage to host microflora, and coevolution with their target bacteria.^24−26^ Phages have also been utilized with various encapsulation delivery systems to improve their antibacterial ability by overcoming the hurdles posed by their host.^27^ Phages immobilized on solid surfaces also contain significant potential in the design of novel antibacterial biomaterials. Covalent attachment of phages on surfaces has been shown to increase the density as well as activity of bound phages.^28−30^ However, covalent immobilization of phages has been attempted on solid surfaces with more focus on the development of phage-based detection systems than antibacterial applications.^28−31^ Covalently attached phages retain their lytic potential and spectrum when immobilized and exhibit higher antibacterial activity as compared to physio-adsorbed phages.^32−34^ Plasma treatment of PDMS surfaces can introduce carboxy groups on the material surface to react with aminosilanes, followed by amine cross-linking of the phages. The resulting phage-immobilized antibacterial surfaces can be adapted for various biomedical applications, including the design of catheter surfaces, microvalves, and implants.^34,35^

This is a proof-of-concept study in which phages are covalently immobilized on NO donor-containing polydimethylsiloxane (PDMS) surfaces (the most widespread polymer used in the fabrication of medical devices). The covalent attachment of antibacterial phages was aimed at improving the clinical applicability of NO-releasing antibacterial and antithrombotic surfaces. NO-releasing surfaces were generated by solvent swelling PDMS in a *S*-nitroso-*N*-acetyl penicillamine (SNAP) tetrahydrofuran solution. PDMS and NO-releasing PDMS surfaces were plasma-activated and treated with aminosilanes, and phages were covalently immobilized on the resulting surfaces via EDC/NHS (1-ethyl-3-(3-(dimethylamino)propyl) carbodiimide/*N*-hydroxysuccinimide) coupling. The phage-containing, NO-releasing materials were evaluated for their antibacterial potential, antithrombotic activity, hemocompatibility, and biocompatibility toward mammalian cells. This is a model and first-of-its-kind study in which active biological antibacterial agents, i.e., phages, were immobilized onto NO-releasing surfaces to control antibiotic-sensitive/resistant bacterial infections and thrombosis on medical-grade indwelling devices.

## 2 Materials and Methods

### 2.1 Materials

Sodium nitrite (NaNO_2_), *N*-acetyl d-penicillamine (NAP), ethylenediaminetetraacetic acid (EDTA), tetrahydrofuran (THF), cell counting kit-8 (CCK-8), 1-ethyl-3-(3-(dimethylamino)propyl) carbodiimide (EDC), *N*-hydroxysuccinimide (NHS), (3-aminopropyl) triethoxysilane (APTMS), phosphate buffer saline (PBS) (10 mM Na_2_HPO_4_, 1.8 mM KH_2_PO_4_, 137 mM NaCl, 2.7 mM KCl, pH 7.4), calcium chloride (CaCl_2_), magnesium chloride (MgCl_2_), sodium chloride (NaCl), sodium citrate, agar, DNAase, glutaraldehyde, Whatman qualitative filter paper grade 2, sodium carbonate (Na_2_CO_3_), sodium bicarbonate (NaHCO_3_), and fluorescein isothiocyanate (FITC) were purchased from the Sigma-Aldrich (St. Louis, MO, USA). Tris base was purchased from Gold Biotechnology (St. Louis, MO, USA). *S*-nitroso-*N*-acetylpenicillamine (SNAP) was purchased from PharmBlock Sciences (USA), inc. (Hatfield, PA, USA). Ultracentrifuge tubes were purchased from Beckman Coulter Life Sciences (Indianapolis, IN, USA). Uranyl acetate was purchased from Honeywell Fluka (New Jersey, NJ, USA). Sylgard 184 silicone elastomer base and Sylgard 184 silicone elastomer curing agent were purchased from Dow Corning Corporation (Midland, MI, USA). Whatman Puradisc 25 mm poly(ether sulfone) syringe filters (0.2 μm), Dulbecco’s Modified Eagle’s Medium (DMEM), and penicillin–streptomycin (5000 U mL^–1^) were purchased from Fischer Scientific (Waltham, MA, USA). SYBR Green was purchased from MedChemExpress (Monmouth Junction, NJ, USA). Luria–Bertani (LB) broth and agar were purchased from Difco Laboratories (Detroit, MI, USA). *Escherichia coli* ATCC 25922 and NIH 3T3 mouse fibroblast cell line (ATCC CRL-1658) were purchased from the American Type Culture Collection (Manassas, VA, USA). Hexamethyldisilazane was purchased from Emsdiasum (Hatfield, PA, USA). Drabkin’s reagent was purchased from Ricca Chemical Company (Arlington, TX, USA). A lactate dehydrogenase (LDH) assay kit was purchased from Roche Life Sciences (Indianapolis, IN, USA). Trypsin–EDTA was purchased from Corning (Corning, NY, USA). Fetal bovine serum (FBS) was purchased from VWR Seradigm Life Sciences (Dublin, Ireland). Calcein AM was purchased from Biolegend (San Diego, CA, USA). Ethidium homodimer III was purchased from Biotium (Fremont, CA, USA).

### 2.2 Phages

#### 2.2.1 Collection of Water Sample for Isolation

The sewage water samples for phage isolation were collected in 50 mL sterile falcon tubes from North Oconee Water Reclamation Facility, Athens, Georgia, USA. The samples were transported in an ice bucket and sediments were removed by centrifugation at 4500 rpm for 10 min. Supernatant from samples was filtered with a 0.2 μm syringe filter and samples were stored at 4 °C for further use.

#### 2.2.2 Isolation and Purification of Phages

*E. coli* ATCC 25922 was chosen as a model host bacterium for phage isolation and subsequent experiments. An overnight culture of *E. coli* was inoculated in the conical flask containing 50 mL of the lysogeny broth (LB) medium and allowed to grow at 37 °C with shaking at 150 rpm. The water sample was mixed with phage buffer (50 mM Tris–HCl, 150 mM NaCl, 10 mM MgCl_2_, 2 mM CaCl_2_, pH 7.5) in a ratio of 1:1 and added to the *E. coli* containing LB medium when the culture reached the logarithmic stage. The mixture of bacteria, water sample, and phage buffer was incubated for 2–3 days at 37 °C with shaking at 150 rpm. After incubation, the suspension was centrifuged at 12,000 rpm for 10 min and the supernatant was filtered with a 0.2 μm syringe filter. The 100 μL of supernatant was mixed with 300 μL of *E. coli* culture in a sterile tube and incubated at 37 °C for 15 min. The incubated mixture was mixed with 5 mL of 0.7% soft agar, overlaid on LB agar plates, and incubated at 37 °C overnight. The next day, plates were observed for phages in the form of clear plaques. For purification of phages, a single plaque containing soft agar was picked and resuspended in 1 mL of phage buffer. The tube containing plaque was vortexed for 2–3 min and centrifuged at 10,000 rpm for 5 min. The supernatant was filtered with a 0.2 μm syringe filter and 100 μL filtrate was incubated with 300 μL of host bacterium for 15–20 min at 37 °C. After incubation, the mixture was mixed with 5 mL of 0.7% soft agar and overlaid on LB agar plates. The plates were incubated overnight at 37 °C to obtain clear plaques. The purification procedure was repeated thrice to obtain pure phage.

#### 2.2.3 Transmission Electron Microscopy

Phages were amplified in LB broth for 5 h and supernatant was collected by centrifuging the bacterial lysate at 12,000 rpm for 10 min. The supernatant was filtered with a 0.2 μm syringe filter, and the filtrate was centrifuged at 28,000 rpm (100,000*g*) for 2 h at 4 °C under vacuum >20 μ (Beckman Optima L-90K Ultracentrifuge, USA). The pellet was resuspended in sterile deionized water, and 5 μL of the phage concentrated suspension was dropped onto carbon-coated copper grids (300 mesh) for 5 min. After 5 min, excess phage suspension was removed using Whatman cellulose filter papers and stained with 2% of freshly prepared uranyl acetate solution for 2 min. Excess stain was removed, and grids were washed thrice with sterile deionized water. Grids were air-dried for 60–90 min and examined under JEOL JEM1011 Transmission Electron Microscope (JEOL USA, Inc., Peabody, MA).

### 2.3 Preparation of SNAP Loaded PDMS Surfaces

PDMS surfaces were prepared by mixing Sylgard 184 base with Sylgard 184 curing agent in a ratio of 10:1.^36^ The mixture of 44 mL was poured into a glass casting plate and allowed to cure overnight in a vacuum oven at 90 °C. On the following day, the PDMS was cut into identical circular samples with 0.635 cm of diameter and 0.225 cm of thickness. The PDMS samples were immersed in SNAP solution (25 mg mL^–1^ in tetrahydrofuran solution) to swell for 24 h with continuous shaking at room temperature. After swelling, the SNAP-PDMS samples were removed and dried overnight at room temperature. The SNAP loaded PDMS samples were immersed in deionized water and sonicated for 5 min to remove surface SNAP crystals. Samples were further dried under vacuum conditions for 24 h. The samples were stored at −20 °C in the dark until used for further experiments.

### 2.4 Surface Modification of PDMS and SNAP-PDMS

Whole phage entities were immobilized onto PDMS and SNAP-PDMS surfaces via EDC/NHS coupling. EDC/NHS coupling was chosen for its ability to immobilize large biomolecules such as phages, retain phage lytic activity, and produce surfaces with high phage density and uniform orientation on solid platforms.^34,37,38^ For incorporation of –OH groups on PDMS and SNAP-PDMS surfaces, plasma treatment was employed because of its applicability in the attachment of various biological molecules such as albumin, lysozyme, and nisin onto polymeric surfaces.^39−41^ Briefly, PDMS and SNAP-PDMS surfaces were treated with low-pressure oxygen plasma treatment for 15 min at 30 W to introduce exposed –OH on the surfaces. Overnight (3-Aminopropyl) trimethoxysilane (APTMS) vapor treatment of plasma-treated PDMS and SNAP-PDMS surfaces was performed to produce aminosilated surfaces, providing a layer of available primary amines for EDC/NHS coupling.

### 2.5 Phage Immobilization

Phages were concentrated from crude bacterial lysates using NaCl-PEG (polyethylene glycol) precipitation method.^42^ Briefly, phage lysate was treated with DNase (0.25 mg mL^–1^) for 1 h at 37 °C to degrade residual host genomic DNA and precipitated by adding NaCl (1M) and PEG 8000 (10% w/v) overnight at 4 °C. The following day, the pellet was recovered by centrifugation at 12,000 rpm for 20 min and resuspended in phage buffer. The phage-PEG suspension was mixed with an equal volume of chloroform and vortexed for 30 s. Organic and aqueous phases were separated by centrifugation at 10,000 rpm for 15 min at 4 °C. The aqueous phase was collected and enumerated for phages using a double-layer agar assay. A high titer phage suspension (∼1 × 10^9^ PFU mL^–1^) was incubated for 15 min with 1 mM EDC and 2.5 mM NHS solution in deionized water containing 2 mM calcium chloride. EDC reacts with carboxyl groups present on the phage surface which is subsequently replaced by NHS to form a stable ester than EDC intermediate. EDC/NHS reacted phages were further incubated with aminated PDMS surfaces for 2 h, to allow immobilization of phages on aminated PDMS surfaces.^38^ After incubation, surfaces were washed five times with PBS (pH 7.4), air-dried in a sterile cabinet (in the dark, 60 min), and stored at −20 °C.

### 2.6 Characterization

#### 2.6.1 Amine Quantification

Aminated PDMS and SNAP-PDMS surfaces were confirmed and quantified using Fluorescein isothiocyanate (FITC) labeling.^43^ Briefly, PDMS, SNAP-PDMS, APTMS treated PDMS and APTMS treated SNAP-PDMS surfaces were incubated with 300 μL of FITC solution (0.001 mg mL^–1^ in 0.2 M carbonate buffer, pH 9) for 18 h in dark environment (*n* = 5). After incubation, the films were washed 5 times with deionized water and sonicated for 30 min to remove the unbound dye, and fluorescent intensity on samples was measured (Ex/Em: 487/528) using a microplate reader (Gen5 Biotek Instruments, USA). The number of amines present on the surfaces was calculated using the standard curve of FITC solution.

#### 2.6.2 Scanning Electron Microscopy and Energy Dispersive X-ray Spectroscopy

Samples (PDMS, SNAP-PDMS, PDMS-Phage, and SNAP-PDMS-Phage) were analyzed using SEM and EDS to evaluate their surface morphology and compositional pattern. All the samples were coated with 10 nm of gold–palladium using a Leica sputter coater prior to imaging. An accelerating voltage of 10 and 20 kV were applied for SEM imaging and EDS analysis, respectively.

#### 2.6.3 SNAP Loading

SNAP loading assays were performed to determine the amount of SNAP loaded in SNAP-PDMS and SNAP-PDMS-Phage surfaces.^36^ Briefly, SNAP-PDMS and SNAP-PDMS-Phage surfaces (*n* = 3) were weighed and submerged in 3 mL of THF with continuous shaking for 24 h at room temperature. The absorbance of extracted SNAP in THF was measured at 340 nm (molar absorptivity = 640 L mol^–1^ cm^–1^) with UV–vis spectrophotometer (Thermo Scientific GENESYS 10S, USA) and correlated with the standard curves of the known SNAP concentrations to determine the total quantity of SNAP impregnated into the PDMS polymer matrix.

#### 2.6.4 Contact Angle Analysis

The static contact angle of all the samples was measured by the sessile drop method using an Ossila contact angle goniometer (Sheffield, UK). A 5 μL of deionized water drop was placed in the center of each sample to avoid imprecise collection of data. The average of ten droplets measured on each of three prepared samples was calculated. Data is reported as the mean ± the standard deviation (*n* = 3).

#### 2.6.5 Phage Density and Distribution Analysis

Phage immobilization on PDMS and SNAP-PDMS surfaces was confirmed by SYBR Green dye staining followed by the imaging using a confocal laser scanning microscope (Zeiss LSM 710 confocal microscope, Germany).^34^ Samples were immersed in 1x SYBR Green solution for 15 min in the dark with continuous shaking. Samples were washed thrice with PBS to remove excess stain and afterward mounted on a glass slide. Samples were imaged using a FITC channel with blue laser (490 nm) using a confocal laser scanning microscope. Based on the fluorescent intensity, the phage count on the PDMS and SNAP-PDMS surfaces was estimated using ImageJ software. Free phage suspension was used as a positive control to determine the fluorescence from free phages. Triplicate samples were used for each sample type and five images were obtained for each sample. The density of phages was calculated for 10 different areas of each sample with average values and standard deviation.

### 2.7 NO Release Kinetics

NO release kinetics of SNAP-PDMS and SNAP-PDMS-Phage surfaces were measured and recorded using Sievers chemiluminescence NO analyzer (NOA 280i, GE analytical, USA). For NO release measurements, SNAP and SNAP-PDMS-Phage samples were submerged in PBS (10 mM, pH 7.4)-EDTA (100 μM) solution in an amber reaction vial to prevent light-induced catalysis of NO. A baseline measurement of the PBS buffer was established prior to loading the sample into the reaction vial. NO release measurements were maintained at 37 °C using a circulating water bath. Released NO from the samples was purged from the reaction vial with a continuous supply of pure nitrogen (flow rate 200 mL min^–1^) to the chemiluminescence detection chamber. The cell pressure of NOA cell ranged from 5.6 to 5.7 and 12.2 to 12.5 Torr for SNAP-PDMS and SNAP-PDMS-Phage samples, respectively. The NO release recorded in ppb was normalized to the surface area of the samples and an NOA constant (mol ppb^–1^ s^–1^) was employed to determine the NO flux (×10^–10^ mol cm^–2^ min^–1^) of analyzed samples. Data was collected over 24 h and samples were stored in 1 mL of PBS at 37 °C between the measurements.

### 2.8 Antibacterial Assays

#### 2.8.1 Plaque Assay for Immobilized Phages

Modified plaque or double layer agar assay was used for rapid assessment of the presence of active phages on the PDMS-Phage and SNAP-PDMS-Phage surfaces. In these experiments, *E. coli* was grown overnight in LB agar to achieve high cell density. PDMS-Phage and SNAP-PDMS-Phage surfaces were washed with sterile PBS five times to remove any surface adsorbed free phages. Washed PDMS-Phage and SNAP-PDMS-Phage discs (0.6 cm diameter) were placed on the agar plates and overlaid with 5 mL of 0.7% soft agar (at 50 °C) containing 300 μL of *E. coli* culture. Once the soft agar had been solidified, plates were incubated at 37 °C for 18–24 h. Active immobilized phages were observed as a clear zone under and around the PDMS-Phage and SNAP-PDMS surfaces. Images were taken to record the activity of immobilized active phages on the PDMS surfaces. Surfaces without phage immobilization, i.e., PDMS and SNAP-PDMS, were used as controls to compare the activity of immobilized phages.

#### 2.8.2 Antibacterial Activity of Immobilized Phages

Immobilized phage activity is dependent on the surface density, orientation, and ability to recognize and infect host bacterium. To determine the antibacterial activity of PDMS, SNAP-PDMS, PDMS-Phage, and SNAP-PDMS-Phage surfaces, the samples were incubated in a growing culture of *E. coli*. Briefly log culture of *E. coli* (OD_600_ = 0.1) was suspended in LB broth and 1 mL of *E. coli* cell suspension was added in each 24-well micro tire plate. Each of the samples was added in a separate well and plates were incubated for 6 h, 37 °C at 120 rpm. Wells containing *E. coli* cell suspensions without any films were termed as control wells. After incubation, plates were removed, and the cell suspension was diluted in PBS (pH 7.4). Each dilution was plated on LB agar and incubated at 37 °C overnight to enumerate the bacterial load in each well. In another similar set of experiments, after completion of the 6 h incubation of the samples containing *E. coli* suspensions 200 μL of sample was aspirated and added to the 96 well plate. The optical density of each treated and control sample was recorded at 600 nm with a microplate reader (Gen5 Biotek Instruments, USA).

Fabricated surfaces were also evaluated for adhesion of bacteria in a growing culture of *E. coli*. As defined earlier, samples were incubated in a log culture of *E. coli* (OD_600_ = 0.1) at 37 °C at 120 rpm for 6 h. After incubation samples were removed, washed with 1 mL of PBS, and homogenized for 60 s at a speed of 2500 rpm in PBS. Samples were vortexed, serially diluted, and plated on LB agar plates. Plates were incubated at 37 °C overnight to determine the number of viable bacteria attached to the surface. For visualization of bacteria on fabricated surfaces, scanning electron microscopy was performed. Briefly, PDMS, SNAP-PDMS, PDMS-Phage, and SNAP-PDMS-Phage surfaces were taken out after 6 h of incubation with bacterial culture. Surfaces were washed with PBS thrice to remove any loosely attached bacteria and fixed with 2% glutaraldehyde solution overnight. The following day, surfaces were dehydrated with a gradient of alcohol (50–100%) for 20 min each. Surfaces were then transferred to hexamethyldisilazane/ethanol (2:1) for 20 min and hexamethyldisilazane/ethanol (1:1) overnight at room temperature to evaporate the contents. The following day, the dried surfaces were mounted on SEM stubs and sputter-coated with a fine layer of palladium–gold. Surfaces were imaged on random spots at varying magnifications.

### 2.9 Platelet Adhesion Assay

All experiments that involve the use of whole blood or its components were approved by the Institutional Animal Care and Use Committee, University of Georgia prior to use. Fresh porcine blood was withdrawn and immediately mixed with 3.2% sodium citrate to prevent clotting. Whole blood was centrifuged at 300 rpm for 12 min and 4000 rpm for 20 min for differential collection of platelet rich plasma (PRP) and platelet poor plasma (PPP), respectively. The concentration of platelets in plasma was determined by an Element HT5 Veterinary Hematology Analyzer (Heska, CO, USA) and the final concentration of 2 × 10^8^ platelets mL^–1^ was adjusted using collected PRP and PPP. Calcium chloride (2.5 mM) was added to platelet solution and samples (*n* = 5) were incubated with 3 mL of platelet solution in rocking condition (25 rpm) at 37 °C for 90 min. After incubation, samples were removed and thoroughly washed with PBS to remove loosely bound platelets. Samples were transferred to microcentrifuge tubes containing 2% triton-phosphate buffer solution (v/v) and incubated for 30 min to achieve complete lysis of adhered platelets. Lactate dehydrogenase released from the lysed platelets was measured using a Roche Cytotoxicity kit, to determine the adhered platelets on surface of each sample. Optical density was measured at 462 nm on microplate reader (Gen5 Biotek Instruments, USA). Platelet adhesion was quantified using calibration curve and compared between samples according to the eq 1, where *P* = platelets cm^–2^
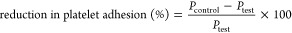
1

### 2.10 Hemolysis Evaluation

A hemolysis assay was performed to assess the hemocompatibility of the fabricated surfaces and followed the NAMSA protocol described by ISO 10993–4.^44^ Briefly, porcine whole blood was collected and immediately mixed with 3.4% sodium citrate to avoid clotting. Collected blood was diluted with calcium and magnesium-free (CMF) PBS to attain a hemoglobulin concentration of 10 ± 1.0 mg mL^–1^. Blood was further diluted with CMF PBS in a ratio of 1:7 in 15 mL conical tubes. Samples (*n* = 5) were immersed in tubes containing diluted blood and incubated at 37 °C for 3 h with periodic inversions. Sterile water and CMF PBS were used as positive and blank controls, respectively, and incubated with tested samples. Tubes were gently inverted at each 30 min interval of incubation. After incubation, tubes were centrifuged at 1900 rpm for 15 min and the supernatant was added with Drabkin’s reagent in a ratio of 1:1. The mixture was incubated for 15 min at room temperature and absorbance was measured at 540 nm with a microplate reader (Gen5 Biotek Instruments, USA). The extent of hemolysis (%) was calculated using the following eq 2, where *A* = Absorbance
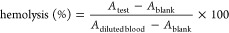
2

### 2.11 Cytocompatibility Studies

#### 2.11.1 Mammalian Cell Culture

Cell cytotoxicity of fabricated surfaces was performed on NIH 3T3 (mouse embryonic fibroblast, ATCC CRL-1658) cell line according to ISO 10993 standards with prior approval from University of Georgia. Cells were cultured in a 75 cm^2^ cell culture flask with Dulbecco’s Modified Eagle Medium (DMEM) with 10% FBS and 1% penicillin–streptomycin. Cell culture flask was incubated at 37 °C with 5% CO_2_ in humified incubator. Cells were allowed to attain the confluency (∼80–90%) and were trypsinized with 0.25% of trypsin supplemented with 5 mM ethylenediaminetetraacetic acid (EDTA). Trypsinized cells were washed with DMEM medium to remove trypsin, counted, and seeded in a 96-well plate (5000 cells per well, 100 μL) in DMEM with 10% FBS and 1% penicillin–streptomycin. Similarly, cells were also seeded in 8-well chambered glass slides (10,000 cells per well, 300 μL) in DMEM with 10% FBS and 1% penicillin–streptomycin. The cells were then incubated at 37 °C with 5% CO_2_ in humified incubator for 24 h.

#### 2.11.2 Cytocompatibility Assay

Cell viability was determined using CCK-8 assay kit as per manufacturer’s guidelines and ISO 10993 standards. Fabricated PDMS, SNAP-PDMS, PDMS-Phage, and SNAP-PDMS-Phage surfaces were incubated in DMEM media with 10% FBS and 1% penicillin–streptomycin for 24 h at 37 °C (1 mg of tested sample per 1 mL of cell culture media). Next day, the extract was collected and contents from each well were aspirated to replace with 100 μL of collected extract in a seeded 96 well plate (*n* = 6). The plate was incubated for 24 h at 37 °C with 5% CO_2_ in humified incubator. After incubation, 10 μL of CCK-8 assay reagent was added to each control and test well. CCK-8 assay reagent contains a water-soluble tetrazolium salt (WST-8: (2-(2-methoxy-4-nitrophenyl)-3-(4-nitrophenyl)-5-(2,4-disulfophenyl)-2*H*-tetrazolium) which is reduced by dehydrogenase of viable cells to form a colored water-soluble formazan dye. The formazan formation was detected at 450 nm after 2 h of CCK-8 reagent exposure. The cell viability (%) was measured relative to treatment control using following eq 3, where *A* = Absorbance
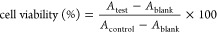
3

For visualization of cell viability, DMEM medium of cells seeded in 8 well chambered glass slides was also replaced with 300 μL of the collected sample extracts. Cells were further incubated for 24 h at 37 °C with 5% CO_2_ in humified incubator. After incubation, the extracts were aspirated, and cells were washed thrice with phosphate buffer saline. Each well was incubated with the mixture of calcein AM and ethidium homodimer III (2 μL each/mL in phosphate buffer saline, 200 μL/well) for 20 min at 37 °C with 5% CO_2_ in humified incubator. Calcein AM is a cell-permeant fluorescent dye that excites and emits upon the activity of esterase which is indicative of metabolically healthy cells whereas ethidium homodimer III, is taken up only by the dead cells when cell loses their cytoplasmic integrity. After incubation, cells were washed with PBS thrice to remove any unbound dye. Cells were visualized under a confocal microscope (Zeiss LSM 710 confocal microscope, Germany) to determine the cellular viability using defined parameters; calcein AM, Ex/Em = 495 nm/515 nm; and ethidium homodimer III Ex/Em = 553 nm/568 nm. Images were captured and analyzed using the Zen system software (Carl Zeiss Canada Ltd., Toronto, ON, Canada).

### 2.12 Statistical Analysis

Data reported are expressed in terms of mean ± standard deviation. One-way analysis of variance was used to calculate the statistical significance and values having *p* < 0.05 were considered as statistically significant. Results were analyzed using GraphPad prism to calculate mean, standard deviation, and *p*-value.

## 3 Results and Discussion

### 3.1 Isolation and Characterization of Phages

*E. coli* phages were isolated and purified from sewage samples. Sewage samples are considered a reliable source of phage isolation due to the vast diversity and abundance of human-associated bacteria present in these contaminated systems.^45^ Double layer agar assays showed the lytic potential of the phages as clear plaques over lawns of *E. coli* were observed (Figure 2A). Plaques were found to be homogeneously uniform in morphology and size (4–5 mm). Morphological analysis of phages showed the icosahedral head having a size of 58.01 ± 2.86 nm with a short tail length and having a total particle size of 76.02 ± 1.28 nm (Figure 2B,C). Based on the morphological analysis, isolated *E. coli* phages can be classified as a family of Podoviridae (order Caudovirales) according to the classification scheme provided by the International Committee on the Taxonomy of Viruses (ICTV).^46^

**Figure 1:**
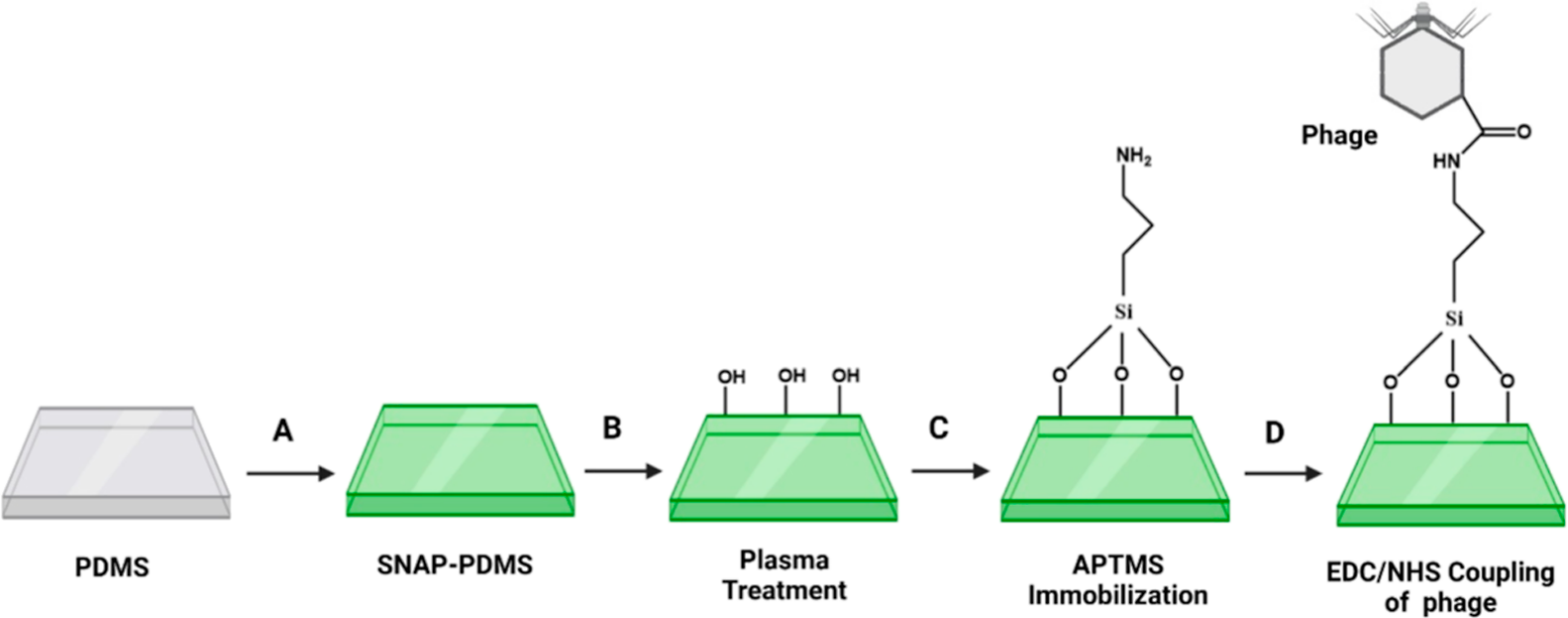
Pictorial representation of phage immobilization on SNAP-PDMS surfaces: (A) PDMS was impregnated using a 25 mg mL^–1^ of SNAP-THF solution to develop the SNAP-PDMS surfaces; (B) SNAP-PDMS films were exposed to oxygen plasma to introduce –OH groups on SNAP-PDMS surfaces; (C) plasma treated SNAP-PDMS surfaces were exposed to APTMS vapors to introduce immobilized amine groups; (D) phages were coupled to aminated SNAP-PDMS surfaces via EDC/NHS coupling.

**Figure 2:**
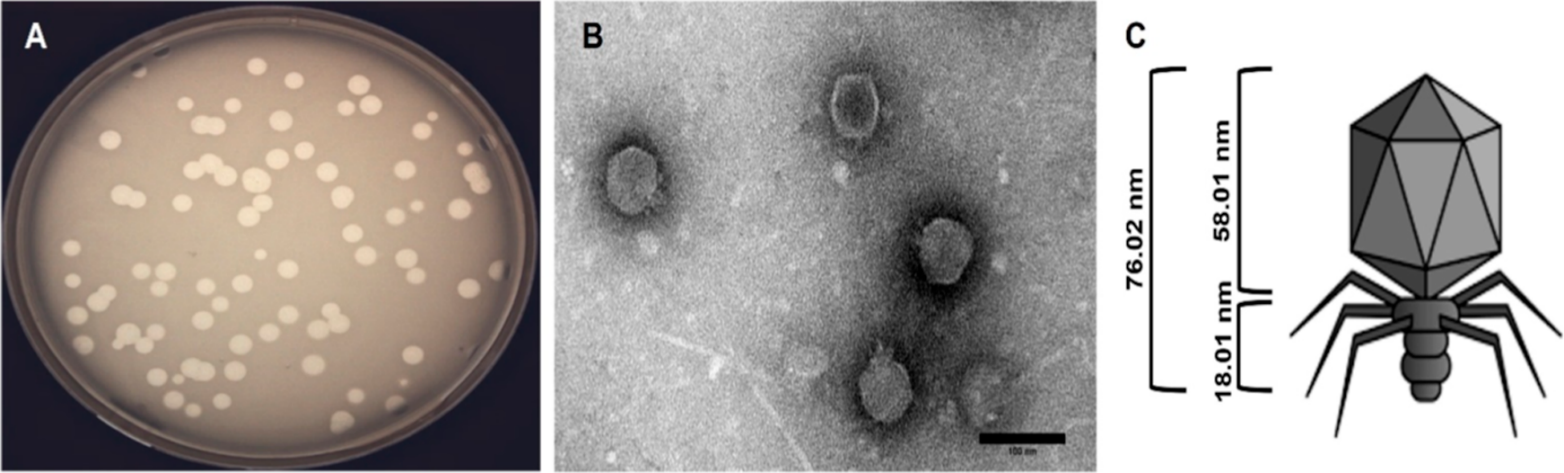
Characterization of *E. coli* phages. (A) Double layer agar assay of phages showing infectivity in terms of clear plaques on *E. coli* host lawn. (B) Morphological analysis of phages by transmission electron microscopy (100 nm scale bar). (C) Illustration figure showing the structure and dimensions of phage, as determined from the TEM images.

### 3.2 Characterization of PDMS-Phage and SNAP-PDMS Surfaces

#### 3.2.1 Fabrication of PDMS-Phage and SNAP-PDMS Surfaces

The present study was aimed to (1) demonstrate the antibacterial activity of covalently immobilized phages on a medical grade polymer surface and (2) use a NO-releasing material as a phage immobilizing platform to explore their antibacterial and antithrombotic potential for clinical applications. Phages as potent antibacterial agents render various advantageous properties over conventional antibiotics, which include self-amplification in the presence of host–pathogen (autodosing), the ability to eliminate antibiotic-resistant bacteria, biocompatibility, nondisruption of host microflora, and low chances of resistance and rapid discovery.^47^ Phage immobilization is a potential strategy to counter colonization of bacteria on medical grade polymers and implants.^32^ Until now, surface immobilization of phages has mostly focused on the bacteria sensing/signal transduction strategies, and limited studies have shown the antibacterial potential of surface-immobilized phages, which ought to be explored for its biomedical implications.^28,31,34^ Phages possess amines and carboxyl groups on their surfaces facilitating their cross-linking with reactive groups containing surfaces.^48^ In the present study, EDC/NHS coupling method was used to immobilize phages, which improves the reaction efficiency, retains phage activity, and produces the reacted surfaces with high phage densities.^34,49^ In earlier studies, carboxyl groups on phages were also activated via EDC/NHS coupling to achieve immobilization on amine functionalized glass and silica surfaces.^28,34,38^

Along with phage immobilization on medical grade polymer PDMS, NO-releasing PDMS surfaces were also employed to design phage-immobilized antibacterial as well as antithrombotic surfaces. SNAP swelling protocol for SNAP loading into PDMS polymer was employed for the fabrication of NO-releasing PDMS surfaces. A 25 mg mL^–1^ SNAP-THF solution was used to swell PDMS for 24 h. THF was chosen as a solvent because it provides rapid solvent evaporation after swelling, excellent loading throughout the polymer, and an excellent solubility limit for SNAP. Using SNAP as the NO donor provides physiological amounts of NO throughout the polymer and significantly increases the hemocompatibility of materials in their in vivo medical applications.^19,50^ In order to immobilize the phages on PDMS and SNAP-PDMS, these surfaces were first treated with oxygen plasma to introduce –OH groups, and then a layer of primary amine was incorporated on these –OH groups via APTMS chemical vapor deposition. EDC/NHS treated phages were used to react with primary amines of plasma/APTMS treated PDMS and SNAP-PDMS surfaces in an aqueous environment.

#### 3.2.2 Amine Quantification

Amine density on PDMS and SNAP-PDMS was calculated via FITC assay, as the presence of amine groups on the surfaces was crucial for phage immobilization. The amines were found to be 2.98 ± 0.59 and 2.08 ± 0.48 nmol amine cm^–2^ for APTMS treated PDMS and SNAP-PDMS surfaces, respectively. Presence of amine groups on APTMS-treated PDMS was also reported to be in a similar range (3.34 ± 0.51 nmol amine cm^–2^) in a recent study.^36^ APTMS exhibits low steric hindrance as compared to 3-aminopropyltriethoxysilane (another commonly used aminosilane for surface aminosilylation), due to having one less methyl group resulting in high aminosilylation.^36^

#### 3.2.3 Scanning Electron Microscopy and Energy Dispersive X-ray Spectroscopy

**Figure 3:**
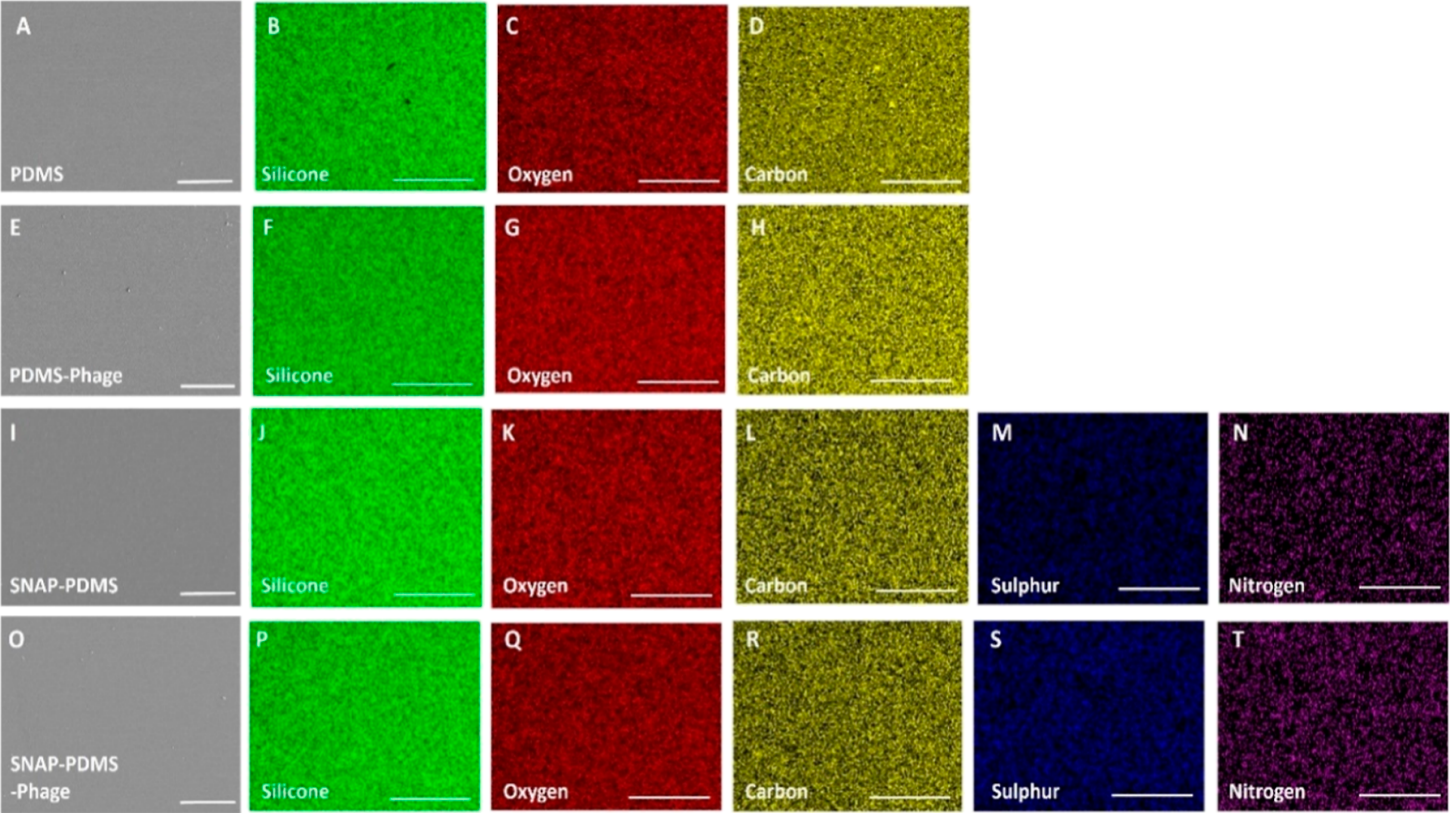
SEM and EDS mapping of PDMS (A–D), PDMS-Phage (E–H), SNAP-PDMS (I–N), and SNAP-PDMS-Phage (O–T). Various elements, i.e., silicone (green), oxygen (red), carbon (yellow), sulfur (blue) and nitrogen (purple), were detected. No significant change in elemental composition was seen after the immobilization of phages on PDMS and SNAP-PDMS surfaces. Scale bar 50 and 250 μm for SEM and EDS images, respectively.

The surface morphology of biomaterials plays a critical role in regulating their interaction with their biological environment, which in turn affects the long-term performance and fate of the biomaterial implants. To examine the surface properties of the phage and nonphage-immobilized biomaterials, SEM was employed (Figure 3). The results show that the incorporation of SNAP does not alter the surface morphology of PDMS, which was also reported in earlier literature.^22,50^ Immobilization of phages on the PDMS and SNAP-PDMS also does not affect the surface morphology of the fabricated materials. To elucidate the elemental composition of the fabricated materials, an EDS analysis was performed. The PDMS surface showed the presence of silicon (green), oxygen (red), and carbon (yellow) as its major constituent elements. PDMS-Phage surfaces also showed similar and consistent elemental composition to the maps generated for the bare PDMS surfaces. SNAP molecules contain sulfur (blue) and nitrogen (purple), along with carbon and oxygen as basic elements. The SNAP-PDMS and SNAP-PDMS-Phage surfaces showed consistent and well-dispersed nitrogen and sulfur in addition to silicon, oxygen, and carbon maps indicating well-dispersed SNAP molecules within the PDMS surface. Results showed that the immobilization of phages does not alter the surface morphology and elemental composition of the PDMS and SNAP-PDMS surfaces.

#### 3.2.4 SNAP Loading

To determine the effect of phage immobilization on the SNAP reservoir of the SNAP-PDMS surfaces, SNAP loading was determined in the SNAP-PDMS and SNAP-PDMS-Phage surfaces. The impregnated SNAP was dissolved out from SNAP-PDMS and SNAP-PDMS-Phage surfaces by incubating samples in excess of THF. The SNAP concentration was found to be similar in both samples at 26.12 ± 3.44 and 27.14 ± 1.99 μg SNAP per mg PDMS for SNAP-PDMS and SNAP-PDMS-Phage surfaces, respectively. Results demonstrate that the phage immobilization process or the presence of phages on the surface does not affect the SNAP reservoir in SNAP-PDMS surfaces. Our results were found to be in concordant with an earlier study, in which the SNAP reservoir was reported to be unaffected by the immobilization of amphotericin on SNAP-PDMS films.^36^

#### 3.2.5 Phage Elution Assay

PDMS-Phage and SNAP-PDMS-Phage surfaces were washed in deionized water to remove the noncovalently bound surface adsorbed phages. The titer of the eluted phages was determined on each step by double layer assay. The residual phages after immobilization were found to be 4.0 ± 1.6 × 10^5^ and 2.2 ± 1.9 × 10^6^ PFU mL^–1^ for PDMS and SNAP-PDMS surfaces, respectively. PDMS-Phage and SNAP-PDMS-Phage surfaces showed the elution of phages on their first and second wash, as shown in Table 1. Subsequent washes for PDMS-Phage and SNAP-PDMS-Phage surfaces did not show any detachment of loosely bound or surface-adsorbed phages. Washed surfaces were found to be active against host bacterial cells showing that the bactericidal effects were ascribed to active immobilized phages rather than any noncovalently bound or inactivated phages.^51^ Our observation indicates that the antibacterial activity of the phage-immobilized surfaces remains consistent, regardless of the number of washes.^52^ Moreover, these robust antibacterial surfaces are ideal for their prolonged clinical applications in moist environments.^53^

**Table 1:** Table Showing Residual and Eluted Phages after the Immobilization Step for PDMS-Phage and SNAP-PDMS-Phage Surfaces

	PDMS-phage surfaces (PFU mL^–1^)	SNAP-PDMS-phage surfaces (PFU mL^–1^)
initial phages	∼1 × 10^9^	∼1 × 10^9^
residual phages	4.0 ± 1.6 × 10^5^	2.2 ± 1.9 × 10^6^
eluted phages wash -1	1.0 ± 6.9 × 10^3^	3.4 ± 2.2 × 10^3^
eluted phages wash -2	3.3 ± 1.6 × 10^2^	1.6 ± 0.9 × 10^2^
eluted phages wash -3–5	not detected	not detected

#### 3.2.6 Contact Angle Measurements

**Figure 4:**
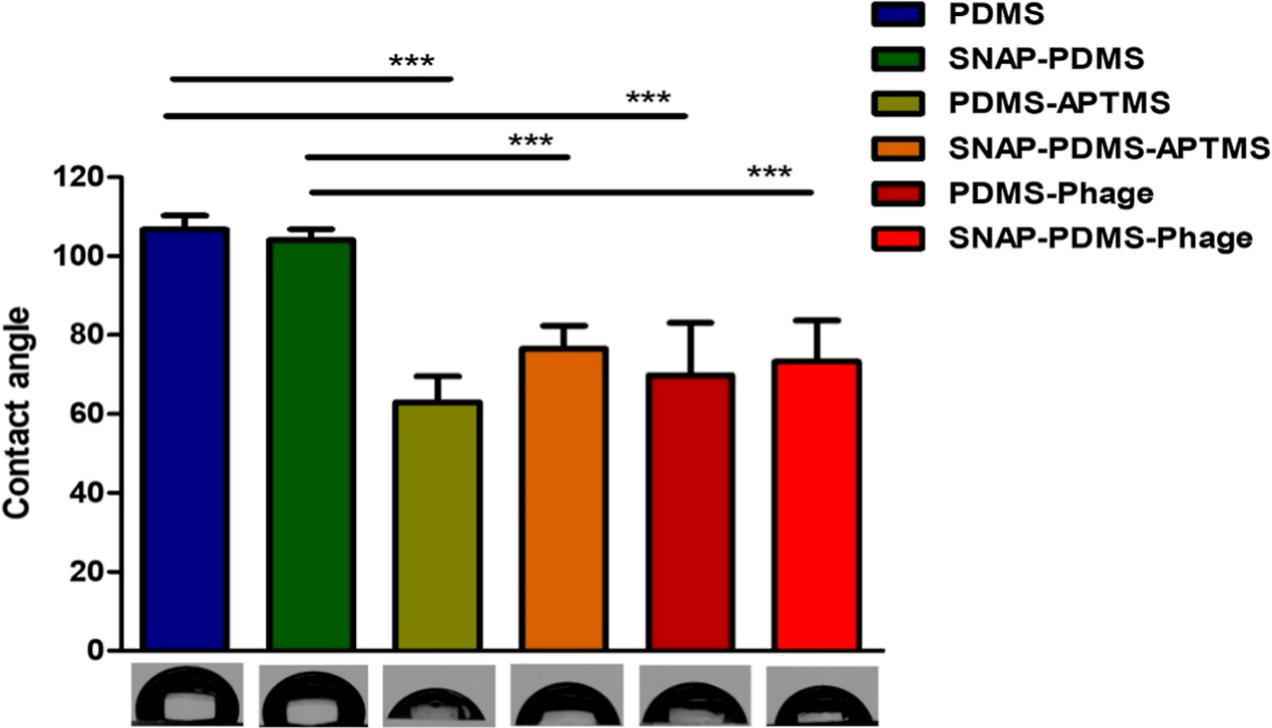
Contact angle of PDMS, SNAP-PDMS, APTMS-treated PDMS, APTMS-treated SNAP-PDMS, PDMS-Phage, and SNAP-PDMS-Phage surfaces. Statistical significance (*p* < 0.001) is indicated by *** and error bars represents the standard deviation of the ten repeated tests for each surface.

Contact angle measurements of the fabricated surfaces were performed to analyze the impact of the fabrication process on surface wettability. Results showed that PDMS has a hydrophobic surface (106.8 ± 3.4°), consistent with earlier reports.^54^ The incorporation of SNAP into PDMS led to a slight decrease in the contact angle of SNAP-PDMS to 104.0 ± 2.0°. Aminosilylation of PDMS and SNAP-PDMS resulted in a significant introduction of hydrophilicity on the surface of these materials. PDMS and SNAP-PDMS surfaces showed significantly decreased contact angle of 62.9 ± 6.6° and 76.5 ± 5.8° after aminosilylation, respectively. The decreased contact angle of APTMS-treated PDMS and SNAP-PDMS surfaces is because of the presence of polar primary amines on the surfaces, which can interact with water molecules. Reduced hydrophobicity of PDMS and SNAP-PDMS surfaces can improve their suitability for clinical use, as hydrophobic materials are highly prone to biofouling via nonspecific protein adsorption.^54^ PDMS-Phage and SNAP-PDMS-Phage surfaces also showed a hydrophilic nature, and their contact angle was found to be 69.8 ± 13.2° and 73.2 ± 10.3° respectively (Figure 4). These results were expected as the immobilization of phages did not alter the hydrophobicity of EDC-NHS treated glass surfaces in an earlier study.^32^

#### 3.2.7 Phage Distribution and Density Analysis

**Figure 5:**
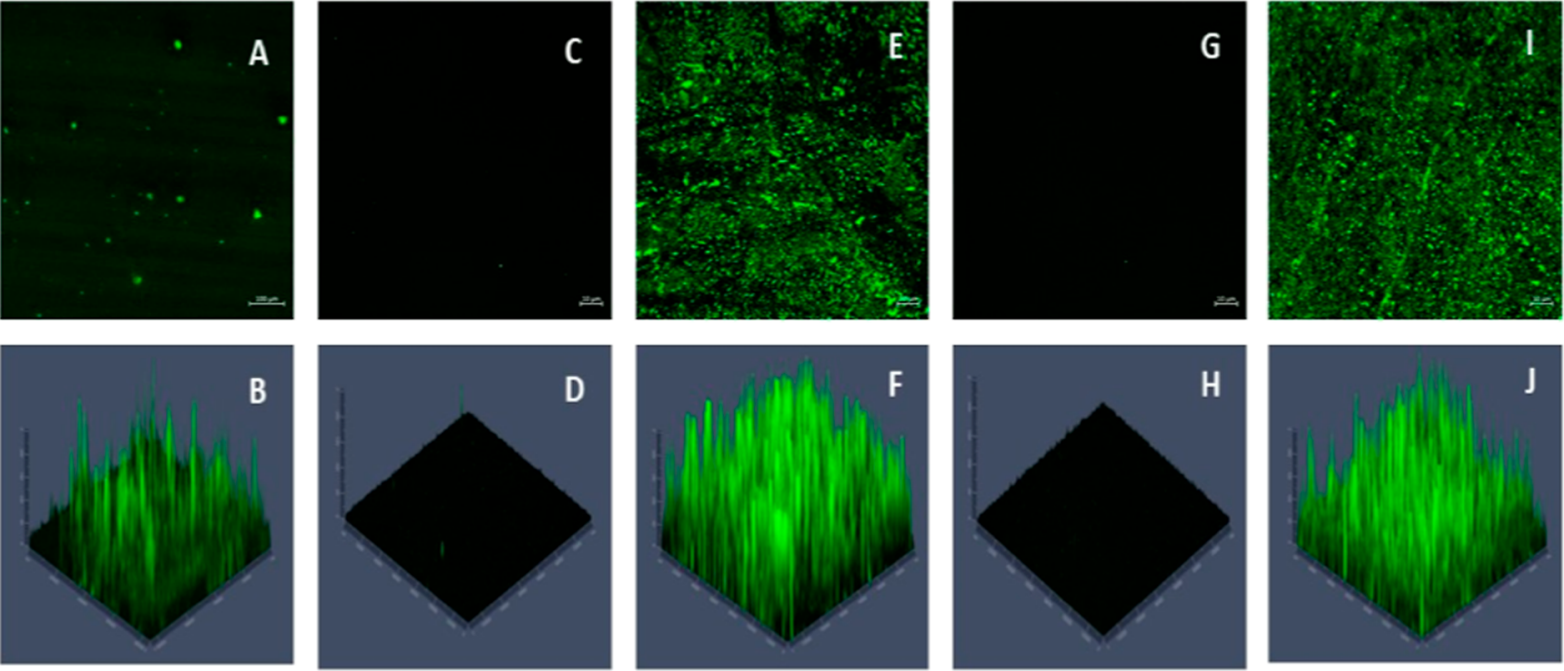
2D and 3D confocal imaging of phages (A,B), PDMS control surfaces (C,D), PDMS-Phage surfaces (E,F), SNAP-PDMS surfaces (G,H) and SNAP-PDMS-Phage surfaces (I,J). Phages bound fluorescent dye showed high signal intensity in phage-immobilized surfaces whereas negligible signal was captured from the bare PDMS and SNAP-PDMS surfaces, establishing successful immobilization of phages on fabricated surfaces.

Phage distribution and density on the surfaces are critical and essential components that regulate these fabricated surfaces’ antibacterial efficacy. Thus, confocal laser scanning microscopy determined and characterized phage immobilization on PDMS and SNAP-PDMS. SYBR Green dye can penetrate the phage capsids and intercalate with phage DNA with high affinity, and this property of SYBR Green was exploited to characterize the immobilized phages.^55^ Free phages labeled with SYBR Green showed fluorescence representing the binding of dye with phage DNA (Figure 5A,B). PDMS and SNAP-PDMS surfaces showed negligible fluorescence under the same staining and imaging conditions (Figure 5C,D,G,H). PDMS-Phage and SNAP-PDMS-Phage surfaces showed fluorescence signals with high intensity (Figure 5E,F,I,J). Based on the fluorescence signal, phages were uniformly distributed on the PDMS and SNAP-PDMS surfaces, suggesting successful and efficient immobilization of phages. Vonsek et al. similarly showed the fluorescence signal of electrostatically immobilized phages on the cellulose fibers, indicating phage immobilization and distribution.^56^ Phage density was also analyzed and found to be 2.4 ± 0.54 and 2.1 ± 0.33 phages um^–2^ on PDMS-Phage and SNAP-PDMS-Phage surfaces, respectively. A slight decrement in phage immobilization on the SNAP-PDMS-Phage as compared to PDMS-Phage surfaces can be explained based on the differences in amine densities of both surfaces. Our results were found to be similar to an earlier study where phage densities were found to be in the range of 0.04 ± 0.01 to 4.25 ± 0.84 phages um^–2^ upon exposure of polyhydroxyalkanoate surfaces with different concentrations of phages.^34^

### 3.3 NO Release Kinetics

**Figure 6:**
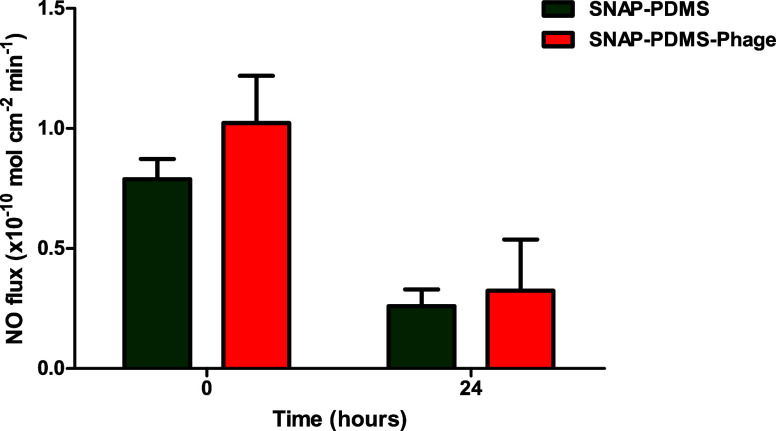
NO release from SNAP-PDMS and SNAP-PDMS-Phage at 37 °C submerged in PBS-EDTA solution. Data is reported in terms of Mean ± SD. No statistical difference was observed between SNAP-PDMS and SNAP-PDMS-Phage surfaces (*p* > 0.05). Bars represent standard deviation.

The NO release kinetics were assessed to evaluate the effect of phage immobilization on NO release from SNAP impregnated PDMS surfaces. NO-containing surfaces previously exhibited potent activity against a number of bacterial pathogens and also possess inhibitory activity for platelet adhesion and activation.^36^ SNAP donors are reported to decompose into disulfide dimers of NAP (or NAP_2_) and NO upon exposure to heat, moisture, light, and metal ions.^57^ To mimic the physiological conditions for NO release from medical grade PDMS polymer, NO release was measured by incubating the sample in 0.01 M PBS with EDTA (100 μM) at 37 °C. Under physiological conditions, SNAP molecules exhibit homolytic cleavage of S–N bonds to release NO via pseudo-first-order kinetics.^58^ SNAP-generated thiol radicals react with residual SNAP to generate more NO molecules.^58^ In the present study, both SNAP-PDMS and SNAP-PDMS-Phage showed a similar and moderate amount of NO release, which has been previously associated with enhanced antibacterial as well as antithrombotic properties in medical grade polymers.^36,50^ The initial NO flux for SNAP-PDMS and SNAP-PDMS-Phage was found to be 0.78 ± 0.08 × 10^–10^ mol cm^–2^ min^–1^ and 1.02 ± 0.19 × 10^–10^ mol cm^–2^ min^–1^ respectively. Both surfaces also showed consistent NO flux after 24 h, as NO release was found to be 0.25 ± 0.06 × 10^–10^ mol cm^–2^ min^–1^ for SNAP-PDMS and 0.32 ± 0.21 × 10^–10^ mol cm^–2^ min^–1^ for SNAP-PDMS-Phage surfaces. The difference in NO release was found to be nonsignificant (*p* > 0.05) for SNAP-PDMS and SNAP-PDMS-Phage surfaces at initial and 24 h time points (Figure 6 and Supplementary Figure S1). The slight, nonsignificant increase in the NO flux in SNAP-PDMS-Phage as compared to SNAP-PDMS surfaces can be explained based on the increased hydrophilicity of the phage-immobilized samples. The increased hydrophilicity may increase the water uptake, resulting in improved NO release, as also previously reported in the literature.^22^ Results showed that the immobilization of phages on SNAP-PDMS surfaces does not significantly affect the release of NO at different time intervals. Moreover, the immobilization of phages on NO-releasing surfaces can be further optimized to design novel materials that not only counter bacterial infection but also inhibit platelet adhesion and activation.

### 3.4 Antibacterial Assays

#### 3.4.1 Plaque Assay for Immobilized Phages

**Figure 7:**
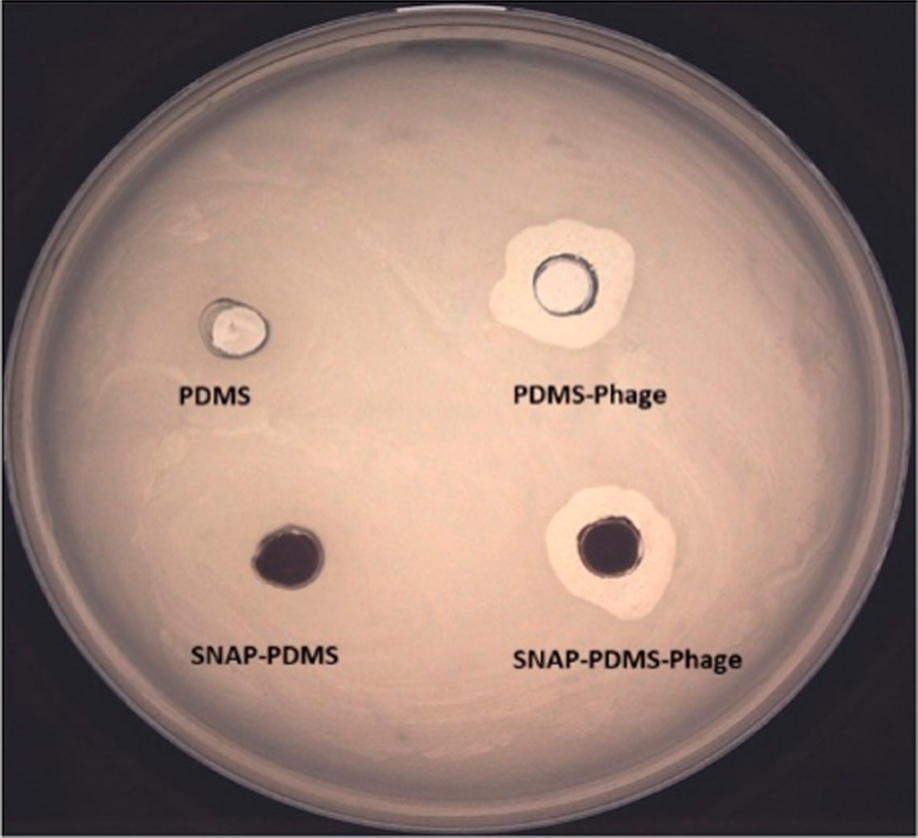
Plaque assay for immobilized phages on PDMS and SNAP-PDMS surfaces against *E. coli*. PDMS-Phage and SNAP-PDMS-Phage surfaces showed bacterial lytic ability, compared to control surfaces.

Immobilization of phages on surfaces does not necessarily ensure that the phages can recognize and kill the target bacterium. The loss of capturing and antibacterial activities of phages may result from improper orientation during immobilization (e.g., parallel immobilization to solid surface or immobilization by tail region, making them inaccessible to host receptors) or loss of the integrity of receptor binding proteins.^53^ Thus, plaque assays for immobilized phages were performed to assess the recognition and killing of the host bacterium. Control PDMS and SNAP-PDMS surfaces do not show any killing of *E. coli* cells. In contrast, PDMS-Phage and SNAP-PDMS-Phage surfaces showed noticeable zones of lysis under and around the materials (Figure 7). The clear zone on soft agar depicts that the phages retain their ability to recognize, attach, and lyse host bacterial cells even after covalent immobilization on PDMS and SNAP-PDMS surfaces. The lysed host cells release new phage progeny which are able to infect and lyse neighboring host bacterial cells to form a clear plaque around the PDMS-Phage and SNAP-PDMS-Phage surfaces.

#### 3.4.2 Antibacterial Activity of Immobilized Phages

To assess the antibacterial effect of immobilized phages, each fabricated surface was separately incubated with growing cultures of *E. coli* in LB broth with shaking conditions for 6 h. Results showed that the immobilization of phages on the PDMS and SNAP-PDMS surfaces drastically confers antibacterial activity against the host bacterium. Control bacterial suspension showed 9.28 ± 0.20 log10 CFU mL^–1^ of bacterial growth, which was comparable with the PDMS treated bacterial suspension (9.51 ± 0.24 log10 CFU mL^–1^). However, the introduction of SNAP into PDMS led to decreased bacterial count (Control vs SNAP-PDMS: 91.57 ± 4.47% of bacterial reduction, *p* < 0.01) which can be ascribed to the antibacterial activity of the NO released from the SNAP loaded in the PDMS surfaces. PDMS-Phage and SNAP-PDMS-Phage surfaces showed significant reduction in bacterial load (Control vs PDMS-Phage: 99.95 ± 0.03% and Control vs SNAP-PDMS-Phage surfaces: 99.99 ± 0.08% of bacterial reduction, *p* < 0.001), as shown in Table 2. A similar decline in the optical density of PDMS-Phage and SNAP-PDMS-Phage surfaces treated bacterial cultures was also seen compared to control surfaces (Supplementary Figure S2).

**Table 2:** Antibacterial Activity of PDMS, SNAP-PDMS, PDMS-Phage, and SNAP-PDMS-Phage Surfaces Materials against Actively Growing *E. coli* Cells

	control	PDMS	SNAP-PDMS	PDMS-Phage	SNAP-PDMS-phage
antibacterial activity against planktonic cells					
mean log10 CFU mL^−1^	9.28 ± 0.20	9.51 ± 0.24	8.30 ± 0.30	5.89 ± 0.11	5.22 ± 0.22
%age reduction vs control group (*p* value)		–69.82 ± 6.50 (*p* > 0.05)	91.57 ± 4.47 (*p* < 0.01)	99.95 ± 0.03 (*p* < 0.001)	99.99 ± 0.08 (*p* < 0.001)
antibacterial activity against adhered cells					
mean log10 CFU cm^–2^		7.66 ± 0.30	6.69 ± 0.20	5.50 ± 0.43	4.93 ± 0.29
%age reduction vs PDMS group (*p* value)			85.79 ± 0.32 (*p* < 0.05)	98.47 ± 2.12 (*p* < 0.001)	99.80 ± 0.05 (*p* < 0.001)

Bacterial attachment on the fabricated materials was also assessed by performing a viable count of bacteria after 6 h of incubation. Results showed that SNAP-PDMS (PDMS vs SNAP-PDMS: 85.79 ± 0.32% of bacterial reduction, *p* < 0.05) showed a significant reduction in the bacterial attachment as compared to the PDMS surfaces. PDMS-Phage (PDMS vs PDMS-Phage: 98.47 ± 2.12% of bacterial reduction, *p* < 0.001) and SNAP-PDMS-Phage (PDMS vs SNAP-PDMS-Phage: 99.80 ± 0.05% of bacterial reduction, *p* < 0.001) surfaces also exhibited a significant decrease in bacterial attachment as compared to PDMS and SNAP-PDMS surfaces, as shown in the Table 2.

**Figure 8:**
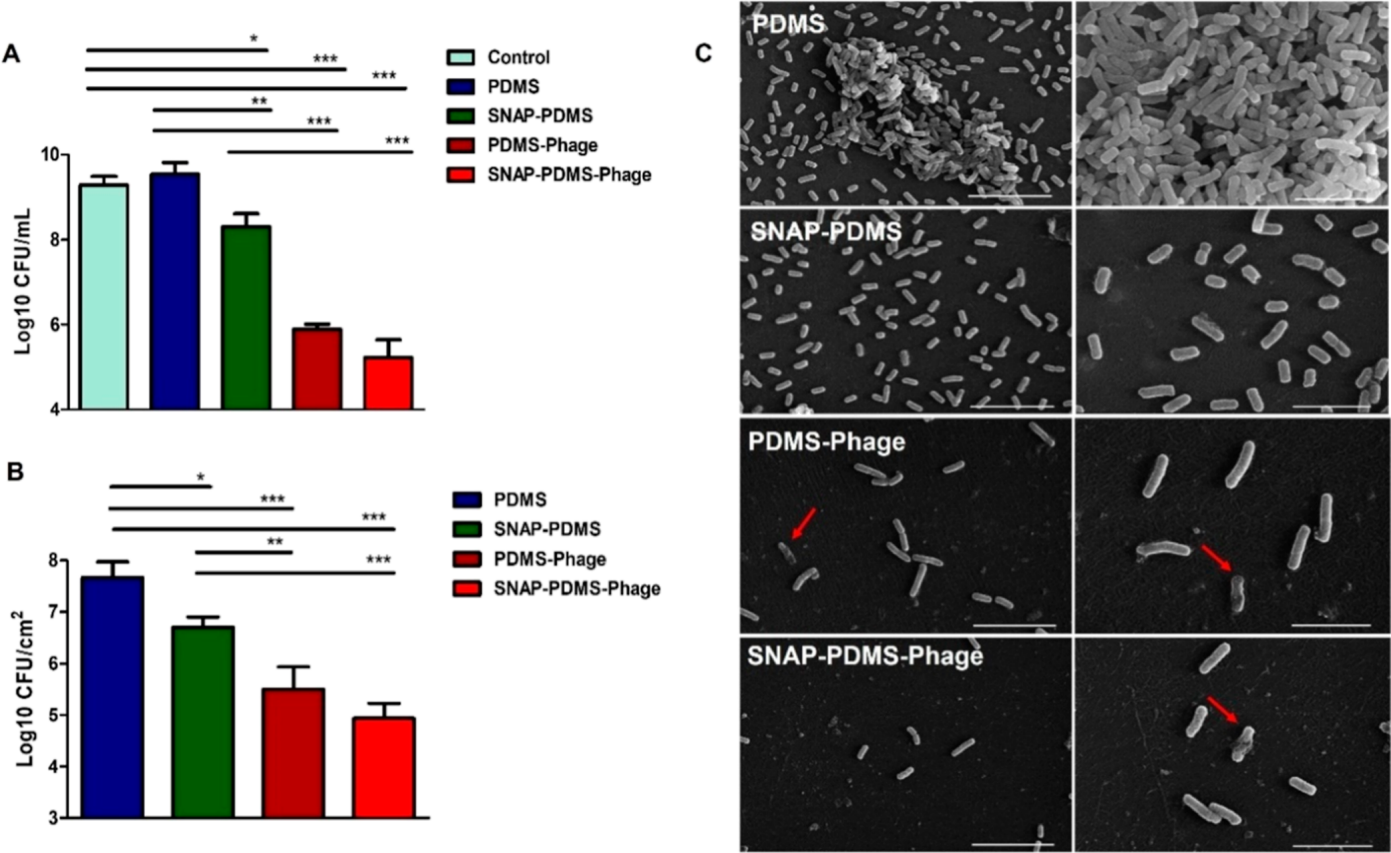
Antibacterial activity of the PDMS-Phage and SNAP-PDMS-Phage surfaces. (A) Fabricated surfaces were incubated with *E. coli* log cultures and compared for the bacterial killing in treated and untreated *E. coli* populations. (B) Antibacterial activity of PDMS-Phage and SNAP-PDMS-Phage surfaces against adhered bacteria in a log culture. (C) SEM images of adhered *E. coli* to PDMS, SNAP-PDMS, PDMS-Phage and SNAP-PDMS-Phage after 6 h of incubation in log culture. Red arrows indicated lysed or damaged bacterial cells (scale bar 10 μm (5000× magnification) and 5 μm (10,000× magnification)). Statistical significance was depicted as * where * corresponds to *p* < 0.05, ** corresponds to *p* < 0.01, and *** corresponds to *p* < 0.001 and bars represent standard deviation.

Scanning electron microscopy of these fabricated surfaces showed a higher degree of bacterial colonization on PDMS followed by SNAP-PDMS, PDMS-Phage, and SNAP-PDMS-Phage surfaces. Biofilm formation was initiated with extracellular matrix production and cell–cell attachment on PDMS surfaces (Figure 8C). However, SNAP-PDMS surfaces showed dispersed bacterial cells on their surfaces, which is attributed to the antibacterial properties of NO released from the surfaces. PDMS-Phage and SNAP-PDMS-Phage surfaces exhibited a low degree of bacterial adhesion on their surfaces which can be correlated with the high antibacterial activity of these surfaces. Phage-induced lysis of bacterial cells (Figure 8C, arrows) on these surfaces led to a decrease in the total population of cells, resulting in a lower degree of adhesion. The phase of the bacterial cells highly dictates the high activity of PDMS-Phage and SNAP-PDMS-Phage surfaces, as the initial log phase provides a high frequency of infection and exponential replication of phages. Results showed that the PDMS-Phage and SNAP-PDMS surfaces are highly efficient in controlling the infection at earlier stages by lysing the host bacterium.

### 3.5 Platelet Adhesion Assay

The benefits of conventional antithrombotic and antiplatelet drugs are irrefutable in reducing indwelling device-associated thrombosis, significantly improving patient outcomes. However, regardless of the type of antithrombotic agent used, there are some serious complications involved in their systemic usage. These primarily include, but are not limited to, thromboembolism, acute hemorrhage, and hypersensitivity reactions.^59,60^ Systemic administration of anticoagulants can lead to adverse effects which may contribute to increased morbidity and mortality, prolonged length of hospital admission, and increased healthcare costs.^61^ The most promising strategy to avoid the adverse effects of systemic usage of anticoagulants is to design novel blood-contacting devices that prevent platelet adhesion and activation on device surfaces.

**Figure 9:**
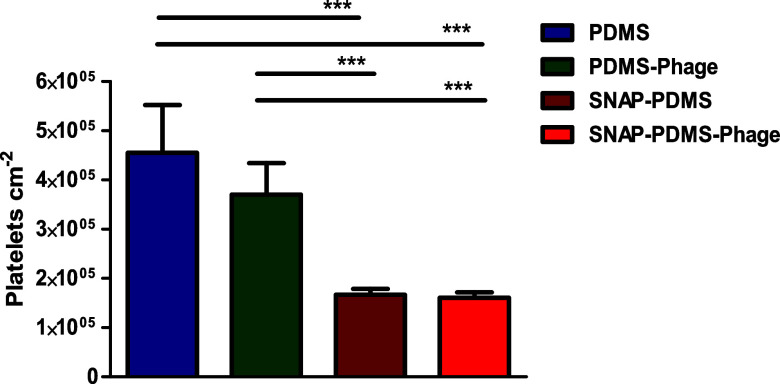
In vitro platelet adhesion of different fabricated surfaces after incubation with porcine platelets. Results were expressed in mean values and bars represent standard deviation. Statistical significance was depicted as *, where *** corresponds to *p* < 0.001.

Platelet adhesion was evaluated to demonstrate the antithrombotic activity of fabricated surfaces, as platelet adhesion is one of the critical factors for thrombosis. PDMS-Phage surfaces showed an 18.67 ± 14.04% reduction in adhered platelets compared to PDMS surfaces (3.70 ± 0.63 × 10^5^ platelets cm^–2^ vs 4.55 ± 0.97 × 10^5^ platelets cm^–2^, *p* > 0.05). The slight decrease in platelet adhesion can be attributed to the increased surface hydrophilicity after aminosilylation and phage immobilization, as shown in the contact angle analysis (Figure 4). Increased hydrophilicity promotes the formation of a monolayer of water molecules on the surface, which increases the thermodynamic requirement needed for foulants to adhere to the surface.^62^ Previous reports have shown that increasing the hydrophilicity results in a decrease in platelet adherence onto polymeric surfaces.^63^ SNAP-PDMS showed a 63.30 ± 2.57% reduction in platelets adhesion compared to control PDMS surfaces (1.66 ± 0.11 × 10^5^ platelets cm^–2^ vs 4.55 ± 0.97 × 10^5^ platelets cm^–2^, *p* < 0.001). SNAP-PDMS-Phage surfaces also exhibited a significant reduction in platelet adhesion of 64.65 ± 2.95% as compared to control PDMS surfaces (1.60 ± 0.10 × 10^5^ platelets cm^–2^ vs 4.55 ± 0.97 × 10^5^ platelets cm^–2^, *p* < 0.001) (Figure 9). Significant reduction in platelet adhesion on SNAP-PDMS and SNAP-PDMS-Phage surfaces against PDMS and PDMS-Phage surfaces can be ascribed to the release of NO, which is comparable with the physiological range of NO flux released by blood vessels.^64,65^ SNAP-PDMS and SNAP-PDMS-Phage surfaces showed similar levels of reduction in platelet adhesion, which demonstrates that the immobilization of phages does not affect the antithrombotic activity of NO-releasing surfaces.

### 3.6 Hemolysis Evaluation

Hemocompatibility is considered one of the critical factors for successful in vivo applications of indwelling blood-contacting medical devices. Improved hemocompatibility increases the life span of these biomaterials by increasing the tolerability and decreasing the blood-associated adverse effects such as thrombus formation.^66^ Interaction of blood and biomaterials may cause rapid lysis of erythrocytes via contact, surface charge, or through the release of toxins, metal ions, and leachates.^67^ Biomaterial-induced hemolysis may exhibit significant deleterious effects on the host’s vascular, renal, myocardial, or central nervous systems causing jaundice, anemia, and other serious pathological conditions.^68^ The hemolytic potential of fabricated materials was tested by incubating samples (*n* = 5) with whole porcine blood for 3 h at 37 °C, using the NAMSA protocol. All the tested fabricated surfaces (PDMS, SNAP-PDMS, PDMS-Phage, and SNAP-PDMS-Phage) were found to be nonhemolytic as the hemolytic activity was found to be 0% when compared to the negative and positive controls (i.e., there was no difference in the absorbance of the samples versus the negative control when reacted with Drabkin’s reagent). The present data is consistent with the data from a previous study in which PDMS and SNAP-PDMS surfaces were reported to be hemocompatible with porcine whole blood in a 2 h study.^36^ Moreover, adding phages is considered a safe choice as they have established their safety and efficacy in treating bacterial infections via intravenous administration for several decades.^69^

### 3.7 Cytocompatibility Evaluation of PDMS-Phage and SNAP-PDMS-Phage Surfaces

**Figure 10:**
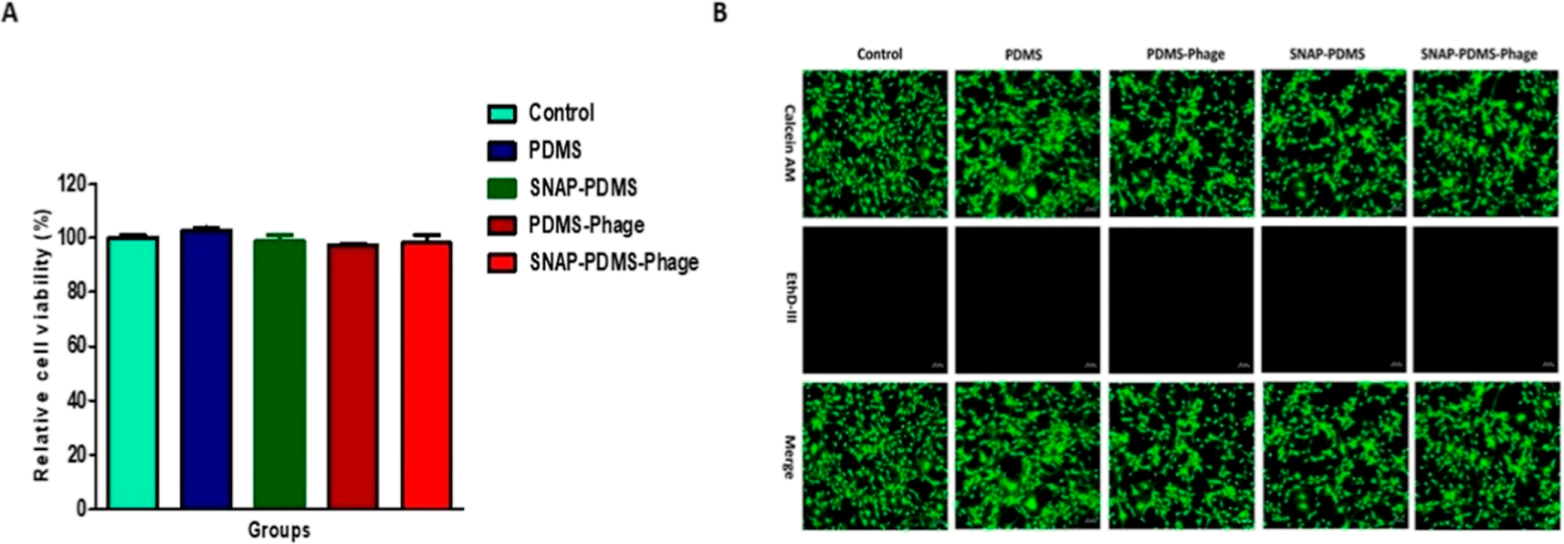
(A) Relative cell viability of the NIH 3T3 mouse embryonic fibroblast cells after 24 h of exposure to leachates of PDMS, SNAP-PDMS, PDMS-Phage, and SNAP-PDMS-Phage surfaces. Error bars represent standard deviation. (B) Live/Dead cell imaging of 3T3 mouse fibroblast cells after treatment with leachates of fabricated surfaces (at 20× magnification, scale bar 50 μm).

To estimate the cytotoxic potential of fabricated biomaterials, NIH 3T3 mouse embryonic fibroblast cells were exposed to leachates of PDMS, SNAP-PDMS, PDMS-Phage, and SNAP-PDMS-Phage surfaces. Cytotoxicity of the leachates from fabricated materials was tested in accordance with ISO-10993. Leachates from the materials were collected in a cell culture medium for 24 h, and 3T3 cells were exposed to the collected leachates for an additional 24 h. Cell response was estimated in terms of cell viability after 24 h of treatment. Figure 10A shows the relative cell viability of fibroblast cells upon treatment with the leachates of the fabricated materials as compared to control. Based on the results of the CCK-8 assay, indirect exposure to the fabricated materials does not exhibit any significant cell cytotoxicity. PDMS and SNAP-PDMS treated cells showed 102.84 ± 1.71% and 98.65 ± 3.23% relative cell viability compared to control cells, respectively. The cytocompatible behavior of SNAP-loaded materials exhibiting physiological NO flux levels was also reported in earlier literature and supports the findings of our present study.^36,57^ Immobilization of phages also does not affect the cytotoxicity profile of the PDMS and SNAP-PDMS surfaces. PDMS-Phage and SNAP-PDMS-Phage treated cells showed 97.13 ± 1.50% and 98.31 ± 5.23% of relative cell viability as compared to control (Supplementary Figure S3). Phages are proteinaceous in structure used in agriculture, medicine, and food processing and reported to be noncytotoxic in earlier studies.^70^ Therefore, the immobilization of phages should not be a considerable factor in modulating the overall cell response of PDMS and SNAP-PDMS materials.

In concordance with the CCK-8 assay, similar cell viability was also observed on Live/Dead staining of cells exposed with leachates of fabricated surfaces. As shown in Figure 10B, all controls and treated cells showed high fluorescence with calcein AM, which is indicative of metabolically active, healthy cells. The ethidium homodimer III signal was found to be negligible because of the inability to permeate live cells and a low number of dead cells in control and treated wells. The results of the present study demonstrate that the SNAP-PDMS-Phage materials are not only potent antibacterial materials but also exhibit biocompatible behavior toward fibroblast cells.

## 4 Conclusion

The present study has presented a proof-of-concept in which active phages were immobilized onto a NO-releasing medical-grade polymer. As these fabricated surfaces exhibit antibacterial as well as antithrombotic properties, the proposed approach shows a high potential to be adapted for the modification and design of medical-grade polymers. In this study, NO-releasing surfaces were fabricated with phages using an EDC/NHS coupling method after aminosilanization. Amine density was found to be 2.98 ± 0.59 and 2.08 ± 0.48 nmol amine cm^–2^ after plasma/APTMS treatment of PDMS and SNAP-PDMS surfaces, respectively. Fabricated surfaces also showed a decrease in the contact angle from 106.8° ± 3.4 (unmodified PDMS) to 73.2° ± 10.3 (SNAP-PDMS-Phage). Phage density was 2.4 ± 0.54 and 2.1 ± 0.33 phages um^–2^ on PDMS and SNAP-PDMS surfaces after immobilization. SNAP-PDMS and SNAP-PDMS-Phage surfaces showed moderate and similar levels of NO release (0.25 ± 0.06 × 10^–10^ and 0.32 ± 0.21 × 10^–10^ mol cm^–2^ min^–1^, respectively) after 24 h of incubation. PDMS-Phage and SNAP-PDMS-Phage surfaces showed antibacterial activity in plaque and log-killing assays. In log killing assays, SNAP-PDMS-Phage surfaces (5.22 ± 0.42 log10 CFU mL^–1^) exhibited high antibacterial activity as compared to PDMS-Phage surfaces (5.89 ± 0.11 log10 CFU mL^–1^), SNAP-PDMS (8.30 ± 0.30 log10 CFU mL^–1^) and PDMS (9.51 ± 0.24 log10 CFU mL^–1^) surfaces. Moreover, bacterial adhesion was found to be lowest in phage-immobilized SNAP-PDMS surfaces compared to other surfaces in bacterial adhesion assay. Along with antibacterial activity, SNAP-PDMS, and SNAP-PDMS-Phage surfaces also showed a significant reduction in surface platelet adhesion compared to control untreated surfaces (63.30% and 64.65%, respectively). All surfaces showed a nonhemolytic nature, as no hemolysis was observed upon incubation with erythrocytes. In addition, none of the fabricated surfaces showed cytotoxicity against fibroblasts postulating biocompatibility for medical applications (>97% cell viability). The results of present study are promising, however the long-term stability and activity of phage-immobilized SNAP-PDMS surfaces in in vitro and in vivo experiments remains to be explored in subsequent studies. In conclusion, this study showed that immobilizing phages on NO-releasing materials could help broaden phage applications in combatting medical device-associated drug-resistant infections and prevent thrombosis on device surfaces.

## Supporting Information Available

Figures; S1: mean NO release profile pattern, S2: graph showing the optical density of bacteria after treatment, S3: Brightfield microscopy images of fibroblast cells after 24 h of exposure, and S4: antibacterial activity of free bacteriophages against host bacterial cells in a 24 h growth assay (PDF)
