# Supplementary material for: Anti-Infective Bacteriophage Immobilized Nitric Oxide-Releasing Surface for Prevention of Thrombosis and Device-Associated Infections
Source: ACS Appl Bio Mater. 2025 Feb 3;8(2):1362–76. doi: 10.1021/acsabm.4c01638 (PMC11836933; doi:10.1021/acsabm.4c01638)
Supplement: Supplementary file 1 — mt4c01638_si_001.pdf [file mt4c01638_si_001.pdf]

Anti-infective bacteriophage immobilized nitric oxide-releasing surface for prevention of thrombosis and device-associated infections.

Supporting Information

Vijay Singh Gondil, [a] Morgan Ashcraft, [b] Sama Ghalei, [a] Anil Kumar, [a] Sarah Wilson, [a] Ryan Devine, [a] Hitesh Handa, [a,b]* and Elizabeth J. Brisbois [a]*

[a] School of Chemical, Materials and Biomedical Engineering, College of Engineering, University of Georgia, Athens, Georgia 30602, United States.

[b] Pharmaceutical and Biomedical Sciences Department, College of Pharmacy, University of Georgia, Athens, Georgia 30602, United States.

Correspondence:

Dr Hitesh Handa and Dr Elizabeth J Brisbois

School of Chemical, Materials and Biomedical Engineering,

College of Engineering, University of Georgia, Athens,

Georgia 30602, United States.

E-mail: HH: hhanda@uga.edu, EJB: ejbrisbois@uga.edu

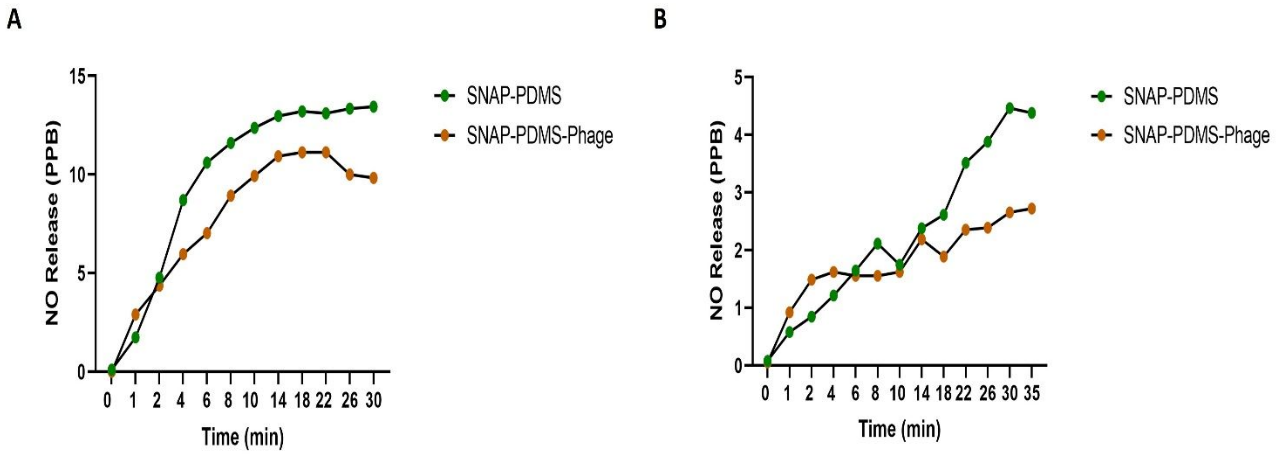

Figure S1: Mean NO release profile pattern (in parts per billion) from SNAP-PDMS and SNAP-PDMS-Phage surfaces at (A) 0 hour and (B) 24 hours.

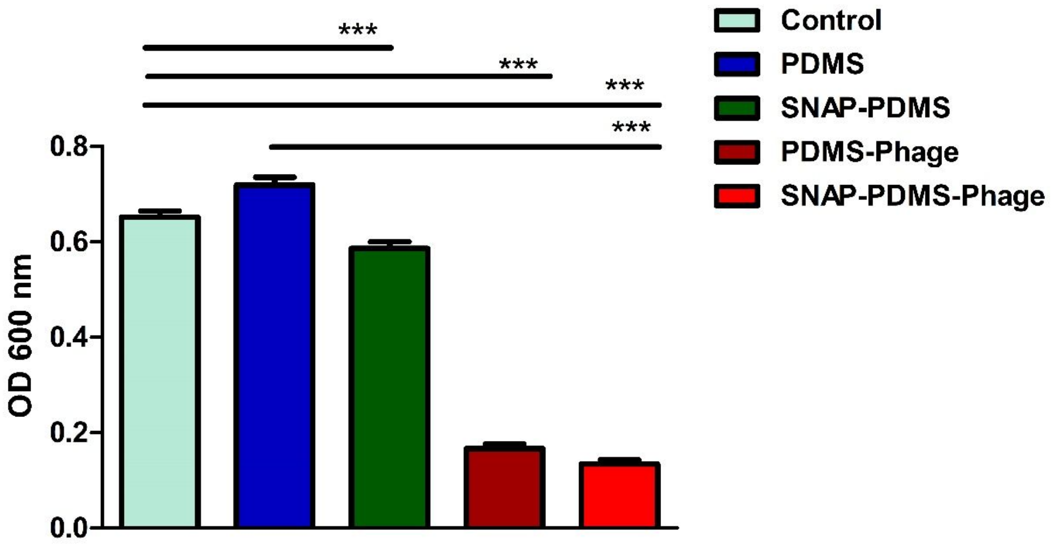

Figure S2: Antibacterial activity of the PDMS-Phage and SNAP-PDMS-Phage surfaces based on the optical density (600 nm) of bacterial cells after 6 hours of treatment in LB media at 37 ℃. Wells without any treatment were termed as control. Statistical significance was depicted as * where *** corresponds to $p < 0.001$ and bars represents standard deviation.

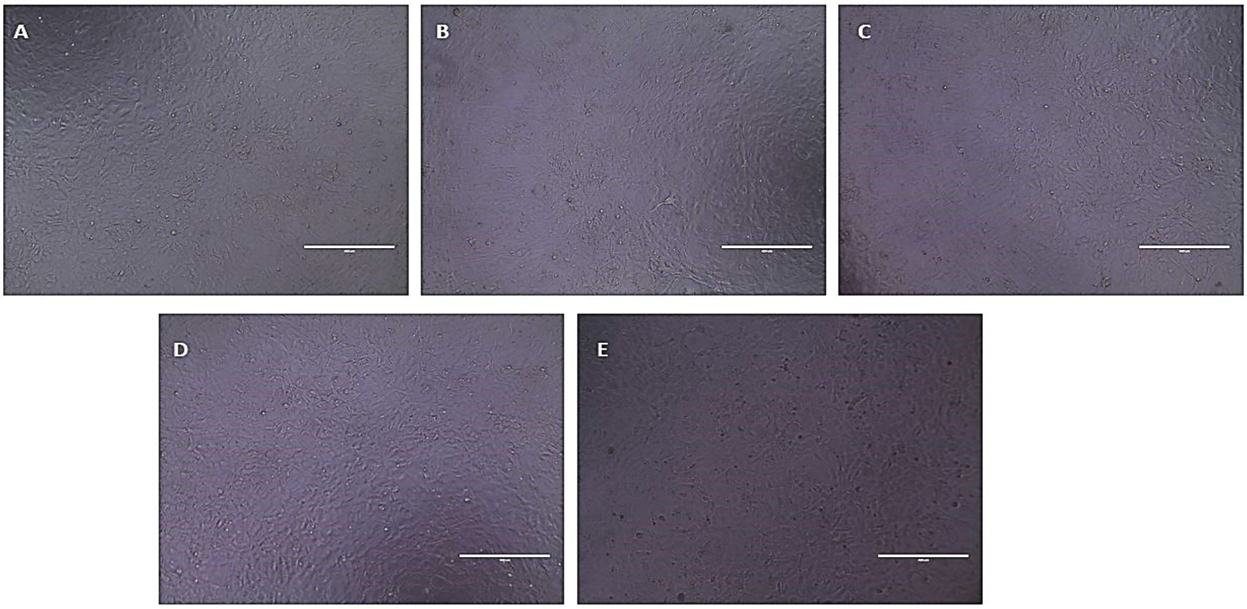

Figure S3: Representative images of the (A) control and cells after treatment with leachates of (B) PDMS, (C) SNAP-PDMS, (D) PDMS-Phage and (E) SNAP-PDMS-Phage surfaces. Confluent cell growth was recorded in control and treatment wells. Scale bar 400 nm.

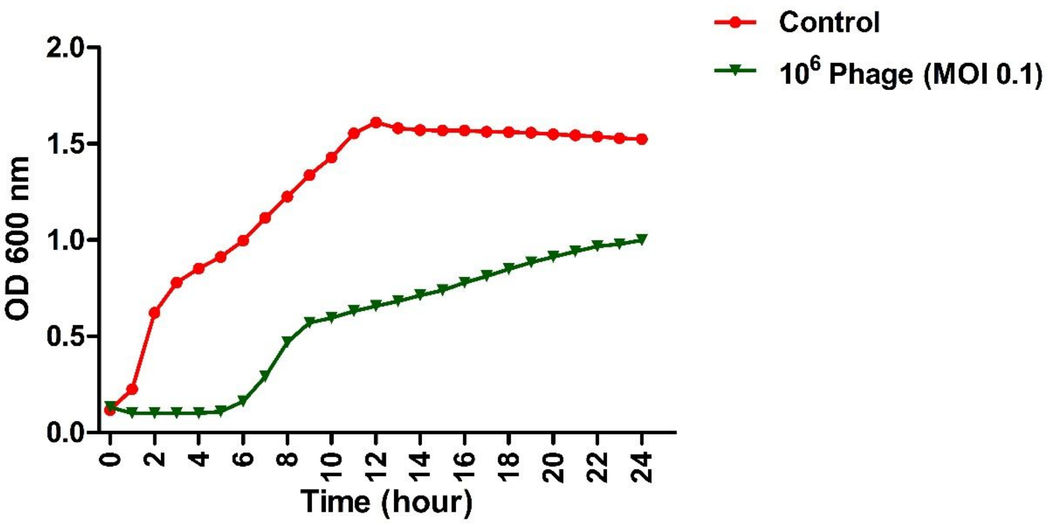

Figure S4: Antibacterial activity of *E. coli* bacteriophages against *E. coli* 25922 host cells in LB medium in a time-kill assay. Cells were infected with bacteriophages at MOI 0.1 at 37 ℃ and optical density (600 nm) was recorded at each hour up to 24 hours.
